# Effectiveness and safety of long-acting aripiprazole 960 mg: preliminary findings from a prospective real-world observational study

**DOI:** 10.1097/YIC.0000000000000635

**Published:** 2026-07-22

**Authors:** Tiziano Prodi, Miriam Olivola, Rodolfo Leuzzi, Monica Biseo, Edoardo Grechi, Nicolaja Girone, Cesare Galimberti, Chiara Di Genova, Kevin La Monica, Federica Calorio, Emma Zuliani, Giada Versaci, Pietro Gallazzi, Norbert Gabriel Kereszteny, Marco Franchini, Andrea Crippa, Gianluca Termine, Roberta Anniverno, Annamaria Cattaneo, Bernardo Dell’Osso

**Affiliations:** a Department of Pharmacological and Biomolecular Sciences, University of Milan; b Department of Mental Health and Addiction Serices, ASST Fatebenefratelli-Sacco; c Department of Mental Health, Department of Biomedical and Clinical Sciences Luigi Sacco, University of Milan, Milan; d Department of Brain and Behavioural Sciences, University of Pavia, Pavia; e ASST Pavia, UOC Oltrepò, Voghera; f Biological Psychiatry Unit, IRCCS Istituto Centro San Giovanni di Dio Fatebenefratelli, Brescia; g “Aldo Ravelli” Center for Neurotechnology and Brain Therapeutic, University of Milan, Milan, Italy; h Department of Psychiatry and Behavioral Sciences, Bipolar Disorders Clinic, Stanford University, Stanford, California, USA; i “Centro per lo studio dei meccanismi molecolari alla base delle patologie neuro-psico-geriatriche”, University of Milan, Milan, Italy

**Keywords:** aripiprazole, long-acting injectable antipsychotics, psychometric assessment, severe mental disorders, treatment compliance

## Abstract

Poor treatment adherence is a major challenge in the management of severe mental disorders, as it is associated with an increased risk of relapse, rehospitalization, symptom worsening, treatment resistance, and all-cause mortality. In 2023 a 2-month 960 mg formulation of aripiprazole 2 months ready-to-use (Ari 2MRTU) was introduced. Compared with monthly long-acting injectable (LAI) drugs, Ari 2MRTU may improve adherence and patient satisfaction by reducing the frequency of healthcare visits. The aim of this prospective longitudinal study was to provide preliminary real-world data on the effectiveness, safety, and acceptability of Ari 2MRTU. Fifty patients, most of whom had previously received aripiprazole, were assessed at baseline, 2 months, and 4 months. Data regarding effectiveness, safety, and acceptability outcomes were collected. Psychometric scores changes were analyzed employing linear mixed-effect regression models accounting for time, age, sex, and previous oral treatment with aripiprazole. Our preliminary findings suggest that Ari 2MRTU is associated with improvements in psychotic symptoms over time, while changes in affective symptoms were less consistent. The findings were also consistent with an overall favorable tolerability profile of Ari 2MRTU. Although adverse events were commonly reported, no unexpected safety signals emerged during the follow-up period, and patients’ attitudes toward LAI treatment generally improved over time.

## Introduction

Treatment of schizophrenia (SCZ) and bipolar disorder is often undermined by low adherence rates to oral treatment (García *et al*., 2016) partly as result of unpleasant side effects and poor illness insight (Habibi Asgarabad *et al*., 2022). Improving adherence is crucial to prevent relapses and rehospitalization, which alter severely the course of the illness, conditioning symptomatic worsening, increased treatment resistance and higher all-cause mortality rates (Correll *et al*., 2026). Recent meta-analysis and reviews showcased that nonadherence reaches around 30% in SCZ (Yaegashi *et al*., 2020) and more than 50% in bipolar disorder (Nierenberg *et al*., 2023).

In addition to the well-established neurotransmitter-based models, growing evidence suggests that glymphatic system dysfunction may represent a complementary pathophysiological mechanism involved in several major psychiatric disorders, including SCZ and bipolar disorder, offering a novel perspective for future translational research (Barlattani *et al*., 2025a, 2025b). Although these mechanisms are still being elucidated, optimizing long-term pharmacological treatment remains the cornerstone of clinical management, highlighting the importance of sustained treatment adherence. Long-acting injectable (LAI) antipsychotics are well-established pharmaceutical strategies that guarantee a more stable plasmatic concentration, with lower peak-to-through fluctuations when compared with oral equivalents (Correll *et al*., 2021). Evidence suggest that LAI antipsychotics are associated with better adherence to medication and health services (Lieslehto *et al*., 2022; Wagner *et al*., 2026) and lower risk of all-cause and nonsuicidal mortality in individuals with SCZ, when compared with oral antipsychotics (Aymerich *et al*., 2025). Moreover, meta-analytic data support that LAIs in bipolar disorder prevent relapse and hospitalization, with fewer hospitalization days (Wagner *et al*., 2026). These findings further support the clinical relevance of treatment adherence in psychotic disorders. Recent evidence from young adults with first-episode psychosis showed that oral antipsychotic treatment at discharge was associated with a higher risk of 1-year readmission, further supporting the role of LAI formulations when clinically appropriate (Besana *et al*., 2024). Moreover, structured pharmacological treatment and systematic clinical monitoring have been identified as core components of specialized, guideline-concordant psychosis care across international recommendations (Scognamiglio *et al*., 2026).

Aripiprazole is a third-generation antipsychotic (TGA) that exerts partial agonism at D_2_ and 5-HT_1_A receptors, and antagonism at 5-HT_2_A receptors, contributing to efficacy across positive, negative, and cognitive symptoms, with a favorable side effect profile (Croxtall, 2012). According to major international guidelines, aripiprazole is approved as first-line treatment for acute and maintenance treatment of SCZ (Keepers *et al*., 2020; McCutcheon *et al*., 2025) as well as acute mania and maintenance treatment of bipolar disorder (Keramatian *et al*., 2023).

Available formulations include oral aripiprazole (OARI) and two LAI options: aripiprazole once-monthly (AOM) and aripiprazole 2 months ready-to-use (Ari 2MRTU).

Previous placebo-controlled studies demonstrated that aripiprazole-IM-depot represents an effective, safe and well-tolerated treatment option for SCZ. Indeed, a 52-week, double-blind, placebo-controlled, randomized and multicenter study in 403 patients diagnosed with SCZ reported improvements in Clinical Global Impressions-Severity of Illness Scale and Positive and Negative Syndrome Scale (PANSS) total scores and a significantly lower rate of relapses at endpoint compared to placebo (10 vs 39.6%). Moreover, it showed that injections had minimal intensity of pain according to the 100-point Visual Analogue Scale (VAS), and the treatment was well-tolerated and safe, with the most common emerging adverse events (occurring in ≥5% of aripiprazole-IM-depot subjects) represented by insomnia, tremor, and headache (Kane *et al*., 2012). Moreover, another randomized, double-blind, multicenter study confirmed the noninferiority of aripiprazole-IM-depot to its oral equivalent in terms of efficacy and tolerability (Xiao *et al*., 2022). Real-Life Assessment of Abilify Maintena, a naturalistic, long-term (12 months), noninterventional cohort study, evaluated the impact of AOM injectable formulation in real-life clinical setting, recruiting 193 patients with a confirmed diagnosis of SCZ from 17 Canadian communities or hospital settings. This study showed significant improvements in global functional status and severity of illness over a 12 months period (Mustafa *et al*., 2019). The use of this depot formulation was evaluated for bipolar disorder as well: in a double-blind, placebo-controlled, 52-week randomized withdrawal study recruiting 266 patients, a reduction in the rate of mood relapses was observed for those individuals in the AOM group compared with placebo, and the treatment was well-tolerated and safe (Calabrese *et al*., 2017).

In 2023 a 2-month 960 mg formulation (Ari 2MRTU 960) was introduced. It represents a recent advancement designed to further reduce treatment burden. This formulation is administered every 56 days and provides plasma exposure comparable to once-monthly aripiprazole (AOM 400), while simplifying dosing schedules (Citrome *et al*., 2023; Wang *et al*., 2024).

The extended dosing interval is the main potential advantage of the bimonthly formulation. Compared with monthly LAIs, Ari 2MRTU reduces the number of administrations, which may translate into improved adherence, fewer healthcare visits, and increased patient convenience and satisfaction. In addition, this reduced frequency may alleviate healthcare system burden, particularly in outpatient settings (Chepke *et al*., 2025). Accordingly, previous findings on three-monthly paliperidone palmitate showed that a reduced injection frequency (four administrations per year) was associated with fewer outpatient visits, without increasing the risk of hospitalization compared with monthly formulations (Cirnigliaro *et al*., 2023).

Pharmacokinetic data support the ability of Ari 2MRTU to maintain stable therapeutic drug concentrations throughout the dosing interval, reinforcing its suitability for maintenance treatment (Harlin *et al*., 2023b). Evidence to date is mainly derived from randomized and open-label clinical studies evaluating safety, tolerability, and pharmacokinetics in clinically stable adult patients with SCZ or bipolar I disorder. These studies have shown that Ari 2MRTU achieves drug exposure comparable to AOM 400 and demonstrates a similar safety and tolerability profile, supporting its noninferiority as a maintenance option (Citrome *et al*., 2023).

However, important limitations remain. Available evidence largely originates from controlled clinical trial settings involving selected and stabilized populations, which may limit generalizability to routine clinical practice. In particular, real-world data on adherence, treatment persistence, and functional outcomes are still scarce. Further observational studies are therefore warranted to better define the effectiveness and clinical utility of Ari 2MRTU in everyday settings.

The aim of this prospective, longitudinal study was to present preliminary findings from a real-world clinical setting of patients treated with Ari 2MRTU, focusing in particular on the effectiveness, safety, and acceptability of such treatment, including treatment adherence and patient satisfaction.

## Methods

Our prospective, longitudinal real-world study included a total of 50 subjects above the age of 18 years and receiving Ari 2MRTU, who were recruited from five Community Mental Health Centers (CMHCs) in ASST Fatebenefratelli-Sacco Hospital, Milan, Italy, and one CMHC located in ASST Pavia, Voghera. No particular exclusion criteria were applied in relation to psychiatric diagnosis and comorbidities or concomitant pharmacological treatment. Diagnosis was established using the Structured Clinical Interview for diagnostic and statistical manual of mental disorders, fifth edition (DSM-5), administered by trained clinicians according to DSM-5 criteria (American Psychiatric Association, 2013). Sociodemographic and clinical data were collected (Table 1). Given the recent introduction of Ari 2MRTU into clinical practice, the present study was conceived as a preliminary exploratory investigation aimed at providing initial insights into its effectiveness, safety, and acceptability. Three main timepoints of clinical evaluation were considered during the study: T0 at baseline, T1 at 2-month follow-up, and T2 at 4-month follow-up. The period of data collection and evaluation was between September 2025 and January 2026. To assess effectiveness and safety, the following psychometric scales were administered and collected:

**Table 1:** Sociodemographic and baseline characteristics

*N*	50
Sex: male (%)	25 (50.00)
Age [median (IQR)]	37.00 (30.00–52.75)
Smoke: yes (%)	28 (56.00)
Alcohol use: yes (%)	16 (32.00)
Family psychiatric history: yes (%)	30/49 (61.22)
Education (%)	
No education	1 (1.96)
Low	12 (23.53)
Medium	31 (60.78)
High	6 (11.76)
Marital status (%)	
Divorced	5 (10.00)
In a relationship	6 (12.00)
Single	39 (78.00)
Job (%)	
Unemployed	26(52.00)
Never worked	2 (4.00)
Employed	18 (36.00)
Retired	4 (8.00)
Housing (%)	
Community	1 (2.00)
Family of origin	20 (40.00)
Own family	8 (16.00)
Alone or with flat mates	21 (42.00)
First diagnosis (%)	
Schizophrenia spectrum and other psychotic disorders	30 (60.00)
Bipolar disorder	12 (24.00)
Obsessive–compulsive disorder	1 (2.00)
Personality disorder	7 (14.00)
Psychiatric comorbidity: yes (%)	20 (40.00)
Behavioral addiction: yes (%)	4 (8.00)
Organic comorbidity: yes (%)	20 (40.00)
Substance use: yes (%)	17 (34.00)
Age at onset (years) [median (IQR)]	22.00 (19.00–30.50)
Age at first therapy (years) [median (IQR)]	23.50 (22.00–35.00)
Duration of untreated psychosis (months) [median (IQR)]	11.00 (0.00–12.00)
History of lack of compliance to pharmacological treatments: yes (%)	41 (82.00)
Previous psychotherapy: yes (%)	19/49 (38.78)
History of psychiatric ward admissions: yes (%)	44 (88.00)
Number of psychiatric ward admissions [median (IQR)]	2.50 (1.00–4.00)
Previous oral treatment with aripiprazole: yes (%)	40 (80.00)
Previous oral treatment with aripiprazole (daily dose in mg) [median (IQR)]	20.00 (15.00–30.00)
Previous oral treatment with aripiprazole (adverse events): yes (%)	2 (4.00)
Previous discontinuation of oral treatment with aripiprazole: yes (%)	23 (46.00)
Previous treatment with monthly aripiprazole: yes (%)	47 (94.00)
Previous treatment with monthly aripiprazole (monthly dose in mg) [median (IQR)]	400.00 (400.00–400.00)
Duration of previous treatment with monthly aripiprazole (months) [median (IQR)]	7.00 (2.75–4.25)
Previous treatment with monthly aripiprazole (adverse events): no (%)	50 (100.00)
Discontinuation of previous treatment with monthly aripiprazole: yes (%)	5 (10.00)
Ongoing oral treatment: yes (%)	23 (46.00)
Ongoing psychotherapy: yes (%)	15 (30.00)
History of involuntary commitment: yes (%)	27 (54.00)
Number of involuntary commitment (median [IQR])	1.00 (1.00–2.00)
Three or more psychiatric ward admissions: yes (%)	25 (50.00)
Two or more involuntary commitments: yes (%)	11 (22.00)
PANSS total score [median (IQR)]	53.50 (41.00–77.75)
PANSS positive symptoms subscale [median (IQR)]	10.50 (7.25–16.25)
PANSS negative symptoms subscale [median (IQR)]	16.00 (11.00–24.00)
PANSS general psychopathology subscale [median (IQR)]	29.00 (22.00–38.75)
MADRS total score [median (IQR)]	11.50 (6.25–17.75)
YMRS total score [median (IQR)]	2.00 (0.00–5.75)

IQR, interquartile range; MADRS, Montgomery–Asberg Depression Rating Scale; PANSS, Positive and Negative Syndrome Scale; YMRS, Young Mania Rating Scale.

1. PANSS: a widely used clinician-administered instrument for assessing symptom severity in SCZ, comprising 30 items divided into three subscales: positive symptoms, negative symptoms, and general psychopathology. It provides both subscale scores and a total score reflecting overall illness severity (Kay *et al*., 1987).
2. Montgomery–Åsberg Depression Rating Scale (MADRS) is a clinician-rated scale designed to assess the severity of depressive symptoms, particularly sensitive to treatment-related changes in mood disorders (Montgomery & Åsberg, 1979).
3. Young Mania Rating Scale (YMRS) is an 11-item clinician-administered scale used to evaluate the severity of manic symptoms, commonly applied in bipolar disorder research and clinical practice (Young *et al*., 1978).
4. Udvalg for Kliniske Undersøgelser (UKU) Side Effect Rating Scale – Patient Assessment of Treatment is a patient-rated instrument assessing the subjective experience of side effects associated with psychotropic medications (Lingjaerde *et al*., 1987).
5. Attitudes toward Depot Treatment Questionnaire (ADTQ) was used to assess patients’ attitudes toward LAI antipsychotic treatment.
6. Drug Attitude Inventory-10 (DAI-10) is a self-report scale assessing patients’ subjective attitudes toward antipsychotic medication, with higher scores indicating more positive attitudes and better adherence likelihood (Hogan *et al*., 1983).
7. The EQ-5D VAS is a standardized measure of self-rated health status, in which patients rate their overall health on a scale from 0 to 100, with higher scores indicating better perceived health (EuroQol Group, 1990).
8. The Compliance Rating Scale (CRS) is a clinician-rated measure of treatment adherence widely used in clinical practice. Patient compliance is assessed using a 7-point Likert scale ranging from 1 (complete absence of compliance) to 7 (excellent compliance).

ADTQ and DAI-10 were administered in their Italian versions. Item wording was translated into English for reporting purposes. UKU data were summarized using predefined thresholds (any adverse event and moderate-to-severe adverse events). For these scales, missing data were handled using complete-case analysis.

Patients provided written informed consent to participate in the study and to allow the use of their anonymized clinical and demographic data for research purposes. The study was conducted in accordance with the Declaration of Helsinki.

### Statistical analysis

Continuous variables were described using the median and interquartile range (IQR), whereas categorical variables were reported as counts and percentages. MADRS, PANSS (positive, negative, general psychopathology, and total scores), and YMRS scores over time were visually depicted using boxplots (Fig. 1).

**Fig. 1:**
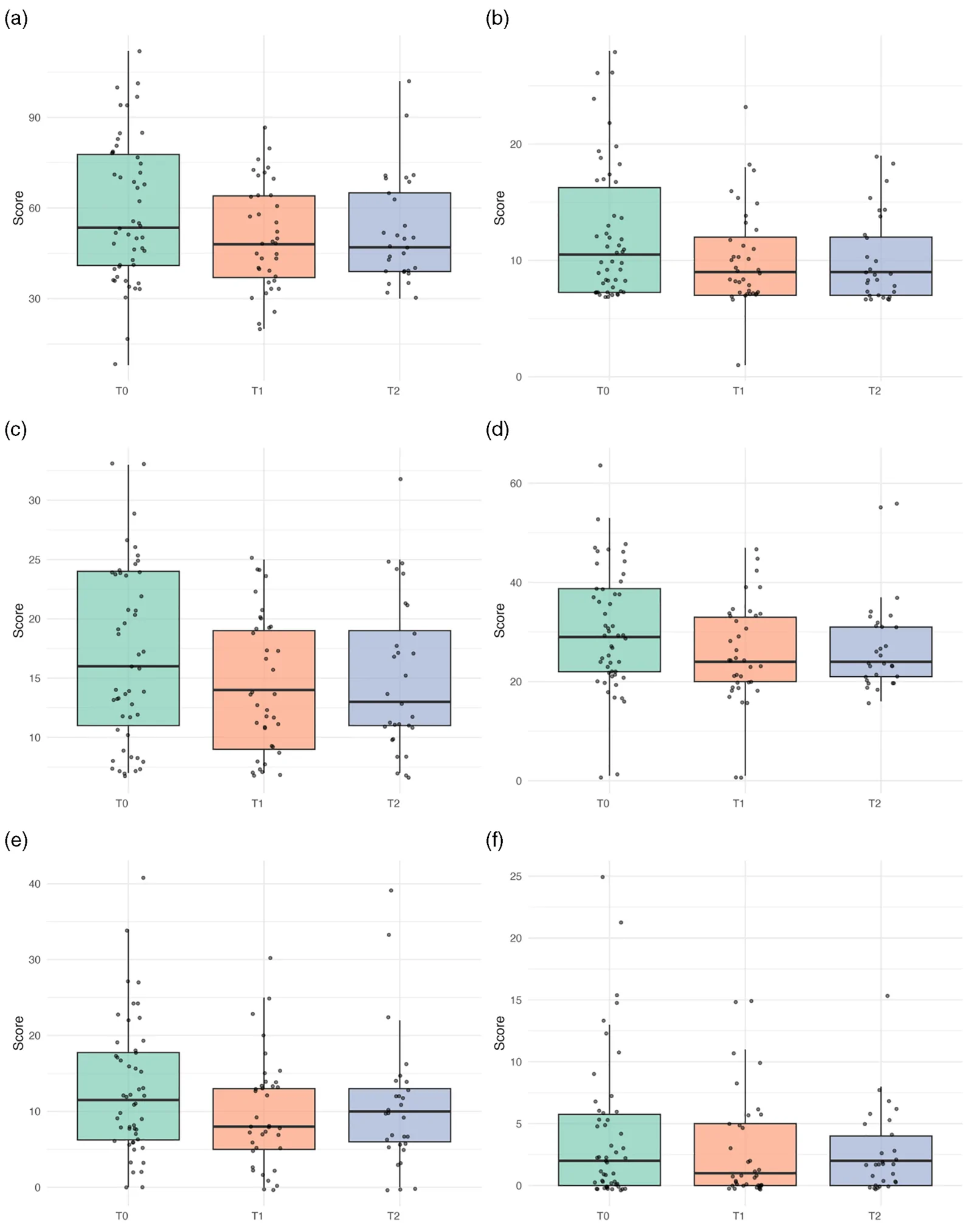
Psychometric scores over time. (a) PANSS total score over time. (b) PANSS positive subscale over time. (c) PANSS negative subscale over time. (d) PANSS general psychopathology subscale over time. (e) MADRS score over time. (f) YMRS score over time. MADRS, Montgomery–Asberg Depression Rating Scale; PANSS, Positive and Negative Syndrome Scale; YMRS, Young Mania Rating Scale.

Linear mixed-effects models (LMER) were then performed to assess whether longitudinal changes in MADRS, YMRS, PANSS-P, PANSS-N, PANSS-G, and PANSS total scores were associated with age, sex, time, and previous oral treatment with aripiprazole. A random intercept was specified to account for within-subject variability. LMER models were chosen as they allow handling of missing data under the assumption of missing at random. Model assumptions were assessed through visual inspection of residual and random effects diagnostic plots. Diagnostic plots showed moderate deviations from normality and some heteroscedasticity at the distribution tails, particularly for PANSS total and YMRS scores; however, no severe violations likely to substantially compromise model interpretation were observed.

All analyses were conducted using RStudio version 4.5.1.

## Results

The total sample consisted of 50 subjects. Forty-one patients were observed at T1, whereas 29 completed the follow-up at T2. Patient follow-up throughout the study is summarized in Fig. 2. Most missing assessments at T1 and T2 reflected ongoing follow-up rather than treatment discontinuation. The median age was 37 (30.00–52.75) years, and 25 (50.00%) were males. Sociodemographic and baseline characteristics are summarized in Table 1.

**Fig. 2:**
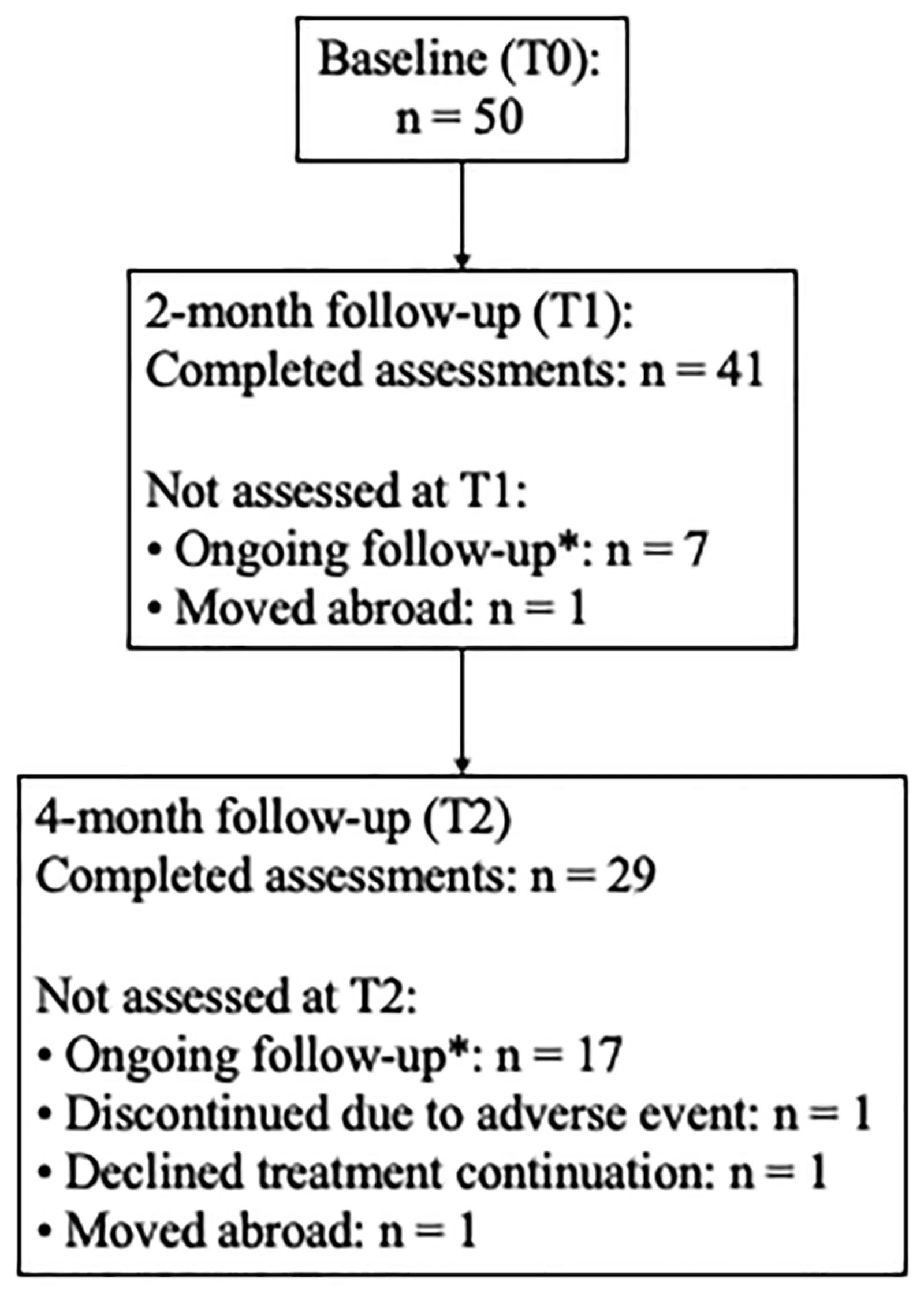
Patient follow-up and assessment availability across study time points. Patients classified as ‘Ongoing follow-up’ had not yet reached the scheduled assessment at the time of data extraction. One additional patient continued treatment but did not complete the psychometric assessments at T1 and T2 and was therefore not included in the longitudinal analyses.

### Effectiveness

#### Positive and Negative Syndrome Scale total score

Median PANSS score at baseline was 53.50 (41.00–77.75), at T1 was 48.00 (37.00–64.00), and 47.00 (39.00–65.00) at T2 (Fig. 1a).

According to the LMER model, PANSS scores showed a significant reduction at both T1 and T2 compared to baseline (*P* < 0.01 both). Complete model estimates and fit indices are reported in the Supplementary Table S1, Supplemental digital content 1, https://links.lww.com/ICP/A168.

#### Positive and Negative Syndrome Scale positive symptoms subscale score

Median PANSS-P score at baseline was 10.50 (7.25–16.25), decreasing to 9.00 (7.00–12.00) at T1, and 9.00 (7.00–12.00) at T2 (Fig. 1b).

According to the LMER model, PANSS-P scores showed a significant reduction both at T1 and T2 compared with baseline (*P* < 0.01 and *P* < 0.001, respectively). Having been treated with OARI in the past was associated with higher PANSS-P scores (*P* < 0.05). Complete model estimates and fit indices are reported in the Supplementary Table S2, Supplemental digital content 1, https://links.lww.com/ICP/A168.

#### Positive and Negative Syndrome Scale negative symptoms subscale score

Median PANSS-N score at baseline was 16.00 (11.00–24.00), at T1 it was 14.00 (9.00–19.00), and 13.00 (11.00–19.00) at T2 (Fig. 1c).

According to the LMER model, PANSS-N scores showed a significant reduction both at T1 and T2 compared with baseline (*P* < 0.05 and *P* < 0.01, respectively). Complete model estimates and fit indices are reported in the Supplementary Table S3, Supplemental digital content 1, https://links.lww.com/ICP/A168.

#### Positive and Negative Syndrome Scale general psychopathology subscale score

Median PANSS-G score at baseline was 29.00 (22.00–38.75), decreasing to 24.00 (20.00–33.00) at T1, and 24.00 (21.00–31.00) at T2 (Fig. 1d).

According to the LMER model, PANSS-G scores showed a significant reduction both at T1 and T2 compared to baseline (*P* < 0.05 and *P* < 0.01, respectively). Complete model estimates and fit indices are reported in the Supplementary Table S4, Supplemental digital content 1, https://links.lww.com/ICP/A168.

#### Montgomery–Åsberg Depression Rating Scale

Median MADRS score at baseline was 11.50 (6.25–17.75), at T1 it was 8.00 (5.00–13.00), and at T2 it was 10.00 (6.00–13.00) (Fig. 1e).

According to the LMER model, MADRS scores showed a significant reduction both at T1 and T2 compared with baseline (*P* < 0.01 and *P* < 0.05, respectively). Complete model estimates and fit indices are reported in the Supplementary Table S5, Supplemental digital content 1, https://links.lww.com/ICP/A168.

#### Young Mania Rating Scale

Median YMRS score at baseline was 2.00 (0.00–5.75); it was 1.00 (0.00–5.00) at T1, and 2.00 (0.00–4.00) at T2. (Fig. 1f).

According to the LMER model, no significant effects of time, age, sex, or previous oral treatment with aripiprazole were observed on YMRS scores. Complete model estimates and fit indices are reported in the Supplementary Table S6, Supplemental digital content 1, https://links.lww.com/ICP/A168.

### Safety

Data on treatment acceptability and self-reported quality of life, assessed using the ADTQ, DAI-10, and EQ-5D, are summarized across timepoints in Table 2. Detailed results of the CRS scale are reported in the Supplementary Table S7, Supplemental digital content 1, https://links.lww.com/ICP/A168. The proportion of patients reporting at least one adverse event and moderate-to-severe adverse events remained overall stable across timepoints (Supplementary Table S8, Supplemental digital content 1, https://links.lww.com/ICP/A168.

**Table 2:** Attitudes toward Depot Treatment Questionnaire, Drug Attitude Inventory-10, and EQ-5D results across timepoints

*N*	T0	T1	T2
	50	41	29
ADTQ, *n* (%)			
1. I prefer taking oral medication daily rather than receiving an injection			
1	10 (20.41)	9 (24.32)	8 (27.59)
2	22 (44.90)	19 (51.35)	10 (34.48)
3	10 (20.41)	5 (13.51)	6 (20.69)
4	1 (2.04)	3 (8.11)	2 (6.90)
5	6 (12.24)	1 (2.70)	3 (10.34)
2. Depot injections are painful			
1	14 (28.57)	11 (29.73)	13 (44.83)
2	11 (22.45)	10 (27.03)	7 (24.14)
3	19 (38.78)	13 (35.14)	7 (24.14)
4	4 (8.16)	2 (5.41)	1 (3.45)
5	1 (2.04)	1 (2.70)	1 (3.45)
3. Depot injections reduce patients’ autonomy			
1	16 (32.65)	15 (40.54)	15 (51.72)
2	17 (34.69)	13 (35.14)	6 (20.69)
3	13 (26.53)	7 (18.92)	7 (24.14)
4	2 (4.08)	2 (5.41)	1 (3.45)
5	1 (2.04)	0 (0.00)	0 (0.00)
4. My doctor did not adequately inform me about the depot option			
1	21 (42.86)	18 (48.65)	19 (65.52)
2	17 (34.69)	9 (24.32)	7 (24.14)
3	7 (14.29)	7 (18.92)	2 (6.90)
4	2 (4.08)	2 (5.41)	1 (3.45)
5	2 (4.08)	1 (2.70)	0 (0.00)
5. Depot injections are helpful to avoid forgetting medication			
1	3 (6.12)	2 (5.41)	3 (10.34)
2	5 (10.20)	2 (5.41)	1 (3.45)
3	14 (28.57)	12 (32.43)	4 (13.79)
4	16 (32.65)	19 (51.35)	17 (58.62)
5	11 (22.45)	2 (5.41)	4 (13.79)
6. I feel more controlled when receiving depot injections			
1	10 (20.41)	11 (29.73)	8 (27.59)
2	11 (22.45)	8 (21.62)	5 (17.24)
3	14 (28.57)	11 (29.73)	7 (24.14)
4	11 (22.45)	6 (16.22)	7 (24.14)
5	3 (6.12)	1 (2.70)	2 (6.90)
7. I believe depot injections reduce the risk of relapse			
1	5 (10.20)	4 (10.81)	2 (6.90)
2	10 (20.41)	5 (13.51)	2 (6.90)
3	13 (26.53)	11 (29.73)	14 (48.28)
4	11 (22.45)	10 (27.03)	8 (27.59)
5	10 (20.41)	7 (18.92)	3 (10.34)
DAI-10, TRUE responses, *n* (%)			
1. Medications help me think more clearly	33 (67.35)	26 (70.27)	24 (82.76)
2. The idea of losing control with medication frightens me	15 (30.61)	14 (37.84)	9 (31.03)
3. Medications improve my life	30 (61.22)	29 (78.38)	23 (79.31)
4. I feel that medications control my thoughts	18 (36.73)	11 (29.73)	7 (24.14)
5. I need medication to stay well	34 (69.39)	28 (75.68)	23 (79.31)
6. Medications are a way to control people	7 (14.29)	6 (16.22)	2 (6.90)
7. The benefits of medication outweigh the side effects	34 (69.39)	26 (70.27)	21 (72.41)
8. Taking medication is a weakness	15 (30.61)	10 (27.03)	3 (10.34)
9. Medications make me feel more like myself	24 (48.98)	19 (51.35)	17 (58.62)
10. Medications make me feel like a failure	11 (22.45)	11 (29.73)	3 (10.34)
EQ-5D Visual Analogue Scale, median (IQR)			
Self-rated health status (0–100)	75.00 (50.00–85.00)	75.00 (60.00–90.00)	77.50 (70.00–90.00)

ADTQ items are rated on a 5-point Likert scale (1 = strongly disagree to 5 = strongly agree); DAI-10 responses are reported as the percentage of ‘TRUE’ answers; EQ-5D scores refer to the Visual Analogue Scale (0–100).

Item wording was translated into English for reporting purposes.

ADTQ, Attitudes toward Depot Treatment Questionnaire; DAI-10, Drug Attitude Inventory-10; IQR, interquartile range.

Overall, these measures suggested a stable or slightly improved pattern of treatment acceptability and self-reported health status over time.

## Discussion

### Sociodemographic and baseline characteristics

For this real-world longitudinal study, 50 patients were recruited. Despite the small sample size and the further reduction in the number of patients assessed 4 months after the start of treatment with Ari 2MRTU (T2), the patients represent real-world clinical cases, predominantly with SCZ spectrum and other psychotic disorders and bipolar disorders. In fact, the frequency of these diagnoses is consistent with the primary therapeutic indications of Ari 2MRTU (Baune, 2024).

Among the sociodemographic characteristics of the sample, there is a balanced distribution by gender (male = 50.00%), smoking habits (smokers = 56.00%), and alcohol consumption (32.00%). Tobacco use is significantly higher among patients with major psychiatric disorders than in the general population, and the percentages in the sample are consistent with recent literature data (Wu *et al*., 2025). Similarly, data on alcohol use also indicate a sample with characteristics comparable to those observed in larger real-world cohorts (Bonner-Reid *et al*., 2025). In the descriptive evaluation of the sample, it was found that 34.00% of patients reported substance use, a prevalence consistent with that observed in major psychiatric disorders (Ward *et al*., 2023). Another finding consistent with the literature is a family history of psychiatric disorders, which is present in 61.22% of patients (Díaz-Castro *et al*., 2021). Regarding clinical course, 88.00% of patients required at least one lifetime hospitalization, with a median of 2.5 admissions over their lifetime (IQR = 1.00–4.00). A total of 54.00% had a history of involuntary commitment, with a median of one involuntary commitment (IQR = 1.00–2.00), 50.00% with three or more psychiatric ward admissions, and 22.00% with two or more involuntary commitments. These findings reflect the chronic and relapsing course of the primary disorders under study, such as SCZ and bipolar disorders, and indirectly suggest the potential reasons underlying the prescription of a LAI antipsychotic (Kishi *et al*., 2021). One of the most significant findings emerging from clinical history was the patient’s poor adherence to the prescribed treatment regimen: 82.00% had a history of lack of compliance to pharmacological treatments. This represents one of the key factors where the administration of a long-acting formulation may exert its greatest clinical impact (Goldberg *et al*., 2025).

Regarding the prescription of aripiprazole, 80.00% of patients in the sample who received Ari 2MRTU had previously been treated with OARI, with a median dose of 20 mg (IQR = 15.00–30.00), and only 4.00% of patients reported adverse events; 94.00% of patients had previously received the monthly intramuscular formulation. This can be interpreted as a possible preference by the prescriber or the patient to initially use a shorter intramuscular formulation in order to assess clinical efficacy and potential adverse effects, following a more gradual pharmacological approach, although current evidence suggests that prior monthly formulations may not be strictly necessary (Citrome *et al*., 2023). However, this prescribing pattern was expected, given the recent introduction of Ari 2MRTU into clinical practice. In routine settings, clinicians are more likely to transition patients who are already clinically stable and have previously demonstrated tolerability to oral or monthly aripiprazole formulations before extending the dosing interval. Consequently, the present sample may not fully represent aripiprazole-naive patients, and caution is warranted when generalizing these findings, particularly those related to safety and tolerability, to such populations.

Finally, only 10.00% of the patients in our sample discontinued the monthly treatment. This finding suggests that while medication adherence plays a pivotal role in the initial decision to prescribe a long-acting formulation, it may not be the decisive factor when transitioning from once-monthly to bimonthly administration. Consequently, the preference for the latter may be guided by other clinical and practical advantages, such as a reduction in injection frequency and a more stable plasma concentration with fewer fluctuations over time (Harlin *et al*., 2023a; Chepke *et al*., 2025). Another reason may be practical: the recent market introduction of the bimonthly formulation has resulted in an expected delay in its real-world adoption. This is further influenced by prescribing inertia, as clinicians often maintain a preference for established formulations with which they have developed extensive clinical experience and confidence (Patel *et al*., 2020).

### Effectiveness

Regarding the analysis of the psychometric scales assessing clinical course at different timepoints, most scales showed significant changes according to regression models.

A closer examination shows that there was a statistically significant reduction in PANSS total scores at both T1 and T2, consistent with the efficacy data reported for AOM (Kim *et al*., 2025).

Evaluating the PANSS subscales, positive symptom scores showed a significant decrease at 2 and 4 months. Age and sex may represent factors associated with differences in PANSS-P scores (Moniem & Kafetzopoulos, 2025). However, according to our findings, those characteristics were not associated with differences in PANSS-P scores. Conversely, a previous oral treatment with aripiprazole was associated with higher PANSS-P scores. It seems plausible that subjects who had assumed OARI were treated for a longer time, which can be an indicator of a more severe illness.

Evaluating the PANSS negative subscale scores, a statistically significant reduction was observed at 2 and 4 months from baseline. Similar findings have also been reported in recent literature (Kobayashi *et al*., 2025), although the extent of the effects of second-generation antipsychotics on primary negative symptoms of SCZ remains highly debated, with a more plausible impact on secondary negative symptoms (Damiani *et al*., 2025).

Indeed, consistent with the pharmacological profile of aripiprazole as a partial dopamine agonist, the PANSS general psychopathology subscale showed a significant reduction at both T1 and T2.

Considering depressive symptoms, a significant reduction in MADRS scores was observed at T1 and T2.

No significant changes were observed in YMRS scores at either timepoint. Nonetheless, no significant worsening of manic symptoms was observed over time. Previous studies have reported reductions in YMRS scores in patients with bipolar disorder treated with AOM (Yang *et al*., 2026). The discrepancy with the present study may be explained by the small sample size, the need for a larger number of patients with bipolar disorder as the primary diagnosis, and the shorter duration of the follow-up timepoints. The absence of significant changes in YMRS scores may also partly reflect a floor effect, given the low baseline manic symptom burden in the study population. However, previous oral treatment with aripiprazole was associated with higher YMRS scores. Similar to what was described for PANSS-P scores, it seems plausible that subjects who had assumed OARI were treated for a longer time, which can be an indicator of a more severe illness. Moreover, previous oral treatment with aripiprazole was not associated with greater symptom improvement during follow-up.

### Safety and acceptability

In the study, scales assessing patients’ attitudes toward medication, and health-related quality of life were administered, and their scores over time were reported (Table 2).

Recent literature indeed highlights the importance of assessing these factors, which influence treatment adherence and the broader concept of health within a biopsychosocial model, as well as documenting, specifically, improvements in these scale scores over time in patients with SCZ spectrum disorders (Aguglia *et al*., 2021).

In our study, a slight increase was observed in the percentage of patients preferring LAI therapy over oral treatment (from T0 = 20.41% to T2 = 27.59%). In addition, the number of patients who strongly disagreed regarding the pain associated with intramuscular injection increased from T0 = 28.57% to T2 = 44.83%. As is well known, this factor substantially impacts the acceptability of long-acting therapies (Franzenburg *et al*., 2025). Similarly, the number of patients who strongly disagreed with the idea that the depot could reduce their autonomy increased (from T0 = 32.65% to T2 = 51.72%).

In the administration of the DAI-10, at 4 months, the percentage of patients who reported thinking more clearly due to the medication increased from 67.35 to 82.76%. In addition, the proportion of patients who believed that the medication could control their thoughts decreased from 36.73 to 24.14%, as did the perception that the medication controls other people, which dropped from 14.29 to 6.90%. Despite the absence of a statistical comparison, the findings mentioned above may reflect changes in patients’ clinical status over time and the generally favorable tolerability profile observed in previous studies of aripiprazole. Nevertheless, these findings should be interpreted with caution, as the number of participants contributing data decreased across follow-up assessments. Therefore, some of the observed improvements may partly reflect differential retention over time rather than exclusively within-subject changes. Accordingly, from T0 to T2, there was an increase in the proportion of patients who believed that the medications improved their lives (from 61.22 to 79.31%). The present findings align with those reported in a recent study, which reported a significant difference in DAI-10 scores between patients receiving LAI treatment and those on oral therapy (Bardi *et al*., 2025). The overall improvement may also be explained by the influence of long-acting aripiprazole on cognitive functions in recent-onset SCZ, as reported by Peitl and colleagues (2021).

Considering the overall quality of life measured with the EQ-5D, the median score slightly increased at T2. Furthermore, a narrowing of the IQR was observed, and – despite the reduction in the sample size at T2 potentially limiting the interpretation of this finding – this may be related to an improvement in the quality of life in those who showed lower scores at previous timepoints. These findings are consistent with previous evidence suggesting that second- and TGAs may have a positive effect on quality of life, while LAI formulations may be associated with more stable improvements in quality of life and a favorable safety and tolerability profile (Sampogna *et al*., 2023).

Regarding adverse events assessed through the UKU, the interpretation of these findings warrants caution. Since UKU assessments were collected from baseline, reported symptoms may reflect previous treatments, concomitant medications, and underlying illness burden, and therefore should not be interpreted as treatment-emergent adverse events specifically attributable to Ari 2MRTU.

### Study limitations

The present findings should be interpreted accounting for some limitations. First, the observational design and the absence of a control group may limit the generalizability of the findings. Second, the relatively small sample size and the lower number of completed assessments at T1 and T2 may affect the interpretation of the findings, particularly those regarding safety measures. As illustrated in Fig. 1, the reduction in available assessments was largely attributable to staggered enrollment and ongoing follow-up at the time of data extraction rather than true treatment discontinuation. Although the LMER models can accommodate missing data under appropriate assumptions, the reduced number of observations at later timepoints may have limited the precision of the longitudinal estimates. Moreover, given the relatively small sample size, the number of covariates included in the mixed-effects models was intentionally limited to avoid model overparameterization. Consequently, residual confounding because of unmeasured clinical variables may have influenced the observed longitudinal trajectories. Third, the duration of the follow-up was just 4 months, limiting the interpretation of both the effectiveness and safety measures for patients undergoing longer treatment duration. Furthermore, the sample included patients with different psychiatric diagnoses, reflecting routine clinical practice and enhancing the real-world relevance of the findings. However, this diagnostic heterogeneity should be considered when interpreting psychometric outcomes, as some measures may be more informative for specific diagnostic groups. Lastly, data were collected in Lombardy, in the areas of Milan and Voghera, limiting the generalizability to patients from other regions.

### Conclusion

Despite some limitations, the present study offers encouraging preliminary results regarding the effectiveness and safety of Ari 2MRTU. We observed that after four months of treatment most psychometric measures showed improvement over the follow-up period. These findings suggest that treatment with Ari 2MRTU may be associated with improvements across several symptom domains in patients with SCZ spectrum disorders. These findings should be interpreted cautiously given the limited sample size and short follow-up. Positive results were also related to the relatively favorable safety profile of Ari 2MRTU. Adverse events were commonly reported, including moderate-to-severe symptoms, but no clinically relevant safety concerns or unexpected adverse reactions were observed. Indeed, UKU assessed subjective side-effect burden at each timepoint, and it is not necessarily related to Ari 2MRTU administration. Nonetheless, an overall improvement in patients’ attitude towards LAI medications was observed.

Further multicentric studies with larger sample sizes should observe patients for a longer follow-up time in order to confirm our encouraging findings.

## Acknowledgements

The authors thank the staff of the Voghera Hospital for their assistance with data collection and subjects recruitment.

Conceptualization: T.P., M.O., and B.D. Methodology: T.P., M.O., and N.G.K. Formal Analysis: T.P. Investigation: T.P., M.O., R.L., and K.L.M. Data curation: T.P., R.L., M.B., C.G., C.D.G., K.L.M., F.C., E.Z., G.V., P.G., N.G.K., M.F., A.Cr., and G.T. Writing – original draft: T.P., R.L., and M.B. Writing – review and editing: T.P. Supervision: R.A., A.Ca., and B.D.

The datasets generated and analyzed during the current study are available from the corresponding author on reasonable request, subject to institutional ethics approval and patient privacy protections. Analysis code (R scripts) is available upon request to facilitate reproducibility.

The study was conducted within routine clinical practice and involved no experimental intervention beyond standard care. All participants provided written informed consent for the use of their anonymized clinical and demographic data for research purposes. The study was conducted in accordance with the Declaration of Helsinki and Good Clinical Practice guidelines.

During the preparation of this manuscript, artificial intelligence tools were used as supportive tools and employed for programming assistance (ChatGPT) and grammar correction (ChatGPT, Grammarly), in full compliance with COPE and ICMJE policies.

### Conflicts of interest

B.D. has received honoraria for lectures from Otsuka and Lundbeck. M.O. has received honoraria for lectures from Otsuka. For the remaining authors, there are no conflicts of interest.

## Supplementary Material
