# Supplementary material for: Effectiveness and safety of long-acting aripiprazole 960 mg: preliminary findings from a prospective real-world observational study
Source: Int Clin Psychopharmacol. 2026 Jul 22;41(6):353–64. doi: 10.1097/YIC.0000000000000635 (PMC13626577; doi:10.1097/YIC.0000000000000635)
Supplement: Supplementary file 1 [file icp-41-353-s001.docx]

**Efficacy and Safety of Long-Acting Aripiprazole 960 mg: Preliminary Findings from a Real-World Longitudinal Study**

**Supplementary Materials**

**Table S1.** LMER model of PANSS total score over time

| **Fixed effects** | **β** | **SE** | **p-value** |
| --- | --- | --- | --- |
| Intercept | 45.0548 | 12.50467 | <.01 |
| T1 | -7.69398 | 2.773762 | <.01 |
| T2 | -9.03448 | 2.628855 | <.01 |
| Age | 0.22161 | 0.218851 | 0.321 |
| Male sex | -0.08085 | 6.182926 | 0.990 |
| Previous oral treatment with aripiprazole | 10.0265 | 6.747158 | 0.150 |

Random intercept for individuals (variance = 234.689, SD = 15.320). Fixed effects coefficients (β) are presented with their standard errors (SE), and p-values. The reference category for sex is “female", and for previous oral treatment with aripiprazole is "no".

Model fit indices were as follows: Akaike Information Criterion (AIC) = 665.628; Bayesian Information Criterion (BIC) = 684.978; log-Likelihood (logLik) = −324.814.

**Table S2.** LMER model of PANSS positive symptoms subscale score over time

| **Fixed effects** | **β** | **SE** | **p-value** |
| --- | --- | --- | --- |
| Intercept | 4.883484 | 3.008539 | 0.117 |
| T1 | -1.76649 | 0.621579 | <.01 |
| T2 | -2.2069 | 0.588878 | <.001 |
| Age | 0.098024 | 0.052699 | 0.075 |
| Male sex | 1.286824 | 1.488258 | 0.395 |
| Previous oral treatment with aripiprazole | 3.74168 | 1.624364 | <.05 |

Random intercept for individuals (variance = 13.874, SD = 3.725). Fixed effects coefficients (β) are presented with their standard errors (SE), and p-values. The reference category for sex is “female", and for previous oral treatment with aripiprazole is "no".

Model fit indices were as follows: Akaike Information Criterion (AIC) = 438.824; Bayesian Information Criterion (BIC) = 458.175; log-Likelihood (logLik) = −211.412.

**Table S3.** LMER model of PANSS negative symptoms subscale score over time

| **Fixed effects** | **β** | **SE** | **p-value** |
| --- | --- | --- | --- |
| Intercept | 10.81556 | 4.629921 | <.05 |
| T1 | -2.21519 | 0.930627 | <.05 |
| T2 | -2.72414 | 0.881547 | <.01 |
| Age | 0.102974 | 0.081125 | 0.216 |
| Male sex | 0.415105 | 2.290686 | 0.858 |
| Previous oral treatment with aripiprazole | 3.390963 | 2.500335 | 0.187 |

Random intercept for individuals (variance = 33.097, SD = 5.753). Fixed effects coefficients (β) are presented with their standard errors (SE), and p-values. The reference category for sex is “female", and for previous oral treatment with aripiprazole is "no".

Model fit indices were as follows: Akaike Information Criterion (AIC) = 502.346; Bayesian Information Criterion (BIC) = 521.696; log-Likelihood (logLik) = −243.173.

**Table S4.** LMER model of PANSS general psychopathology subscale score over time

| **Fixed effects** | **β** | **SE** | **p-value** |
| --- | --- | --- | --- |
| Intercept | 29.35689 | 6.242544 | <.001 |
| T1 | -3.72836 | 1.478932 | <.05 |
| T2 | -4.10345 | 1.402241 | <.01 |
| Age | 0.020711 | 0.109154 | 0.851 |
| Male sex | -1.79505 | 3.08508 | 0.566 |
| Previous oral treatment with aripiprazole | 2.894643 | 3.365976 | 0.398 |

Random intercept for individuals (variance = 57.165, SD = 7.561). Fixed effects coefficients (β) are presented with their standard errors (SE), and p-values. The reference category for sex is “female", and for previous oral treatment with aripiprazole is "no".

Model fit indices were as follows: Akaike Information Criterion (AIC) = 565.507; Bayesian Information Criterion (BIC) = 584.858; log-Likelihood (logLik) = −274.754.

**Table S5.** LMER model of MADRS score over time

| **Fixed effects** | **β** | **SE** | **p-value** |
| --- | --- | --- | --- |
| Intercept | 12.27735 | 6.202843 | 0.059 |
| T1 | -3.33492 | 1.226038 | <.01 |
| T2 | -3 | 1.161286 | <.05 |
| Age | 0.03898 | 0.108704 | 0.723 |
| Male sex | -2.76094 | 3.069185 | 0.377 |
| Previous oral treatment with aripiprazole | 1.394233 | 3.350209 | 0.681 |

Random intercept for individuals (variance = 59.657, SD = 7.724). Fixed effects coefficients (β) are presented with their standard errors (SE), and p-values. The reference category for sex is “female", and for previous oral treatment with aripiprazole is "no".

Model fit indices were as follows: Akaike Information Criterion (AIC) = 545.636; Bayesian Information Criterion (BIC) = 564.987; log-Likelihood (logLik) = −264.818.

**Table S6.** LMER model of YMRS score over time

| **Fixed effects** | **β** | **SE** | **p-value** |
| --- | --- | --- | --- |
| Intercept | -1.78108 | 3.046457 | 0.564 |
| T1 | -0.6711 | 0.617926 | 0.282 |
| T2 | -1.13793 | 0.585363 | 0.057 |
| Age | 0.072894 | 0.053374 | 0.184 |
| Male sex | -0.01349 | 1.507178 | 0.993 |
| Previous oral treatment with aripiprazole | 3.58614 | 1.645085 | 0.039 |

Random intercept for individuals (variance = 14.296, SD = 3.781). Fixed effects coefficients (β) are presented with their standard errors (SE), and p-values. The reference category for sex is “female", and for previous oral treatment with aripiprazole is "no".

Model fit indices were as follows: Akaike Information Criterion (AIC) = 438.833; Bayesian Information Criterion (BIC) = 458.183; log-Likelihood (logLik) = −211.416.

**Table S7.** CRS score over time

|  |  | **T0** | **T1** | **T2** |
| --- | --- | --- | --- | --- |
| N (%) | | 50 | 41 | 29 |
| **CRS score** | **Compliance level** |  |  |  |
| 2 | Poor compliance | 1 (2.00) | 0 (0.00) | 0 (0.00) |
| 3 | Partial compliance | 9 (18.00) | 2 (5.41) | 0 (0.00) |
| 4 | Moderate compliance | 3 (6.00) | 2 (5.41) | 2 (6.90) |
| 5 | Good compliance | 9 (18.00) | 12 (32.43) | 8 (27.59) |
| 6 | Very good compliance | 18 (36.00) | 12 (32.43) | 12 (41.38) |
| 7 | Excellent compliance | 10 (20.00) | 9 (24.32) | 7 (24.14) |

The Compliance Rating Scale (CRS) is a 7-point Likert scale ranging from 1 (complete absence of compliance) to 7 (excellent compliance).

**Table S8.** Reported adverse event assessed using the UKU-PAT scale

| N (%) | **T0** | **T1** | **T2** |
| --- | --- | --- | --- |
| Any reported adverse events | 44 (89.80) | 28 (75.68) | 23 (82.14) |
| Moderate-to-severe adverse events | 35 (71.43) | 18 (48.65) | 19 (67.86) |

Moderate-to-severe adverse events were defined as a UKU-PAT score ≥ 2.
